# Vital Signs: Noise-Induced Hearing Loss Among Adults — United States 2011–2012

**DOI:** 10.15585/mmwr.mm6605e3

**Published:** 2017-02-10

**Authors:** Yulia I Carroll, John Eichwald, Franco Scinicariello, Howard J. Hoffman, Scott Deitchman, Marilyn S. Radke, Christa L. Themann, Patrick Breysse

**Affiliations:** ^1^Office of Science, National Center for Environment Health, CDC; ^2^Division for Toxicology and Human Health Services, Agency for Toxic Substances and Disease Registry, Atlanta, Georgia; ^3^National Institute on Deafness and Other Communication Disorders, National Institutes of Health, Bethesda, Maryland; ^4^Office of the Director, National Center for Environment Health, CDC; ^5^Division of Emergency and Environmental Health Services, National Center for Environment Health, CDC; ^6^ National Institute for Occupational Safety and Health, CDC.

## Abstract

**Introduction:**

The 2016 National Academies of Sciences report “Hearing Health Care for Adults: Priorities for Improving Access and Affordability” included a call to action for government agencies to strengthen efforts to collect, analyze, and disseminate population-based data on hearing loss in adults.

**Methods:**

CDC analyzed the most recent available data collected both by questionnaire and audiometric tests of adult participants aged 20–69 years in the 2011–2012 National Health and Nutrition Examination Survey (NHANES) to determine the presence of audiometric notches indicative of noise-induced hearing loss. Prevalence of both unilateral and bilateral audiometric notches and their association with sociodemographics and self-reported exposure to loud noise were calculated.

**Results:**

Nearly one in four adults (24%) had audiometric notches, suggesting a high prevalence of noise-induced hearing loss. The prevalence of notches was higher among males. Almost one in four U.S. adults who reported excellent or good hearing had audiometric notches (5.5% bilateral and 18.0% unilateral). Among participants who reported exposure to loud noise at work, almost one third had a notch.

**Conclusions and Implications for Public Health Practice:**

Noise-induced hearing loss is a significant, often unrecognized health problem among U.S. adults. Discussions between patients and personal health care providers about hearing loss symptoms, tests, and ways to protect hearing might help with early diagnosis of hearing loss and provide opportunities to prevent harmful noise exposures. Avoiding prolonged exposure to loud environments and using personal hearing protection devices can prevent noise-induced hearing loss.

## Introduction

Hearing plays an important role in communication, health, function, and quality of life. Hearing loss is the third most common chronic physical condition in the United States and is twice as prevalent as diabetes or cancer (*1*).

Untreated hearing loss is associated with decreased social, psychological, and cognitive functioning. Hearing ability is inversely associated with distress, somatization, depression, and loneliness among all age groups (*2*,*3*). The economic cost to society of age-related hearing loss has been estimated to be $297,000 over the lifetime of every affected person. Hearing loss is associated with low employment rates, lower worker productivity, and high health care costs. Adults with hearing loss are more likely to have low income and be unemployed or underemployed than adults with normal hearing (*2*,*3*). Nationally, the total cost of first-year hearing loss treatment is projected to increase fivefold between 2002 and 2030, from $8.2 billion to $51.4 billion (*4*).

Noise is the most common modifiable environmental cause of hearing loss among young and middle-aged adults, and the most common self-reported cause of hearing loss among men (*5*). In 2014, an estimated 21.0% of adults aged ≥18 years had difficulty following a conversation amid background noise, 11.2% had tinnitus (i.e., the perception of ringing in the ears or other sounds such as buzzing, hissing, and clicking), and 5.9% had sensitivity to everyday sounds (hyperacusis).* In addition to hearing loss, chronic exposure to noise has been associated with increased stress, anxiety, depression, blood pressure, heart disease incidence, distractibility, annoyance, tinnitus, hyperacusis, and other health problems (*6*).

The 2016 National Academies of Sciences report included a call to action for government agencies to strengthen efforts to collect, analyze, and disseminate population-based data on hearing loss in adults (*2*). CDC analyzed data from the 2011–2012 National Health and Nutrition Examination Survey (NHANES) to estimate the prevalence of audiometric notches and exposure to noise among adults aged 20–69 years.

## Methods

NHANES^†^ is a continuous, cross-sectional health interview and examination survey designed to assess the health and functional status of the civilian, noninstitutionalized U.S. population. The 2011–2012 NHANES cycle included audiometric testing and hearing-related questions for a nationally representative sample of adults aged 20–69 years. Using the standard NHANES audiometric protocols, audiograms were analyzed using an algorithm (*7*) to identify high-frequency audiometric notches that suggest hearing loss caused by exposure to noise. An audiometric notch is a deterioration in the hearing threshold (the softest sound a person can hear). This study defined the presence of a high-frequency audiometric notch when any threshold at 3, 4, or 6 kHz exceeded the average threshold at 0.5 and 1 kHz by ≥15 decibel (dB) hearing level (HL) and the 8 kHz threshold was at least 5 dB HL lower (better) than the maximum threshold at 3, 4, or 6 kHz. Statistical analyses were weighted as recommended for NHANES data. Logistic regression was performed to evaluate the notch prevalence among age groups and its association with sociodemographic factors (sex, race/ ethnicity, education, income) and exposure to noise. NHANES 2011–2012 defined “loud” noise as when “you had to speak in a raised voice to be heard,” and “very loud” as when “you have to shout in order to be understood by someone standing 3 feet away from you.”

## Results

During NHANES 2011–2012, a total of 3,583 participants aged 20–69 years had complete audiometric data (response rate 76.6%, among 4,677 participants who completed household interviews). The weighted prevalence of an audiometric notch among U.S. adults aged 20–69 years was 39.4 million or 24.4% (6.2% bilateral notch and 18.2% unilateral notch) (Table 1).

**TABLE 1:** Percentages of adults aged 20–69 years with an audiometric notch* in one ear (unilateral notch) or both ears (bilateral notch), by selected characteristics — National Health and Nutrition Examination Survey, United States, 2011–2012

Characteristic (No.)	Bilateral or unilateral notch		Bilateral notch		Unilateral notch	
	% (SE)	OR (95% CI)	% (SE)	OR (95% CI)	% (SE)	OR (95% CI)
**Overall (3,583)**	24.4 (1.73)	—	6.2 (0.57)	—	18.2 (1.32)	—
**Sex**						
Male (1,841)	31.6 (1.89)	Referent	8.6 (0.76)	Referent	23.0 (1.53)	Referent
Female (1,742)	17.0 (1.90)	0.44 (0.35–0.56)	3.7 (0.95)	0.35 (0.19–0.66)	13.3 (1.23)	0.48 (0.40–0.57)
**Age group (yrs)**						
20–29 (803)	19.2 (2.34)	Referent	4.2 (1.31)	Referent	14.9 (1.95)	Referent
30–39 (721)	24.9 (2.95)	1.40 (0.98–2.00)	4.6 (0.84)	1.16 (0.50– 2.67)	20.4 (2.65)	1.47 (0.99–2.17)
40–49 (682)	29.0 (2.86)	1.72 (1.28–2.31)	7.70 (1.31)	2.07 (1.05–4.09)	21.3 (2.21)	1.62 (1.22–2.16)
50–59 (715)	27.3 (2.05)	1.58 (1.04–2.42)	8.7 (1.56)	2.27 (0.92–5.56)	18.7 (2.21)	1.39 (0.86–2.24)
60–69 (662)	20.6 (2.99)	1.09 (0.66–1.82)	5.3 (0.91)	1.28 (0.52–3.16)	15.3 (2.76)	1.04 (0.59–1.85)
**Race/Ethnicity**						
White, non-Hispanic (1,240)	24.0 (2.08)	Referent	6.5 (0.67)	Referent	17.6 (0.67)	Referent
Black, non-Hispanic (996)	21.1 (1.54)	0.85 (0.64–1.13)	3.6 (0.47)	0.54 (0.35–0.82)	17.5 (1.62)	0.96 (0.72–1.28)
Mexican American (381)	31.8 (3.12)	1.48 (1.06–2.05)	11.1 (2.63)	1.93 (1.09–3.42)	20.6 (2.21)	1.31 (0.94–1.83)
**Education**						
Less than high school (690)	29.7 (3.91)	1.49 (1.00–2.21)	8.1 (2.09)	1.75 (0.89–3.42)	21.6 (3.24)	1.41 (0.92–2.15)
Completed high school (737)	28.4 (2.87)	1.40 (1.10–1.77)	8.4 (0.86)	1.78 (1.26–2.51)	20.0 (2.43)	1.28 (0.98–1.68)
More than high school (2,156)	22.1 (1.63)	Referent	5.1 (0.60)	Referent	17.0 (1.24)	Referent
**Poverty income ratio**						
≤1 (848)	22.9 (1.81)	1.17 (0.87–1.58)	5.3 (0.80)	0.90 (0.49–1.62)	17.6 (1.62)	1.29 (0.92–1.80)
>1 to <5 (1,876)	27.0 (2.02)	1.46 (1.13–1.89)	6.7 (1.01)	1.20 (0.65–2.23)	20.3 (1.31)	1.57 (1.14–2.17)
≥5 (607)	20.2 (1.94)	Referent	6.1 (1.19)	Referent	14.1 (1.98)	Referent
**Self-reported work exposure to noise^†^**						
No (2,360)	19.9 (2.04)	Referent	5.1 (0.73)	Referent	14.8 (1.53)	Referent
Yes (1,223)	32.6 (2.48)	1.95 (1.40–2.72)	8.2 (1.09)	1.91 (1.17–3.11)	24.4 (2.20)	1.96 (1.37–2.81)
**Self-reported hearing status^§^**						
Excellent or good (2,953)	23.5 (1.92)	Referent	5.5 (0.63)	Referent	18.0 (1.57)	Referent
Little, moderate, or a lot of trouble hearing (626)	28.3 (2.99)	1.29 (0.91–1.82)	9.0 (1.53)	1.73 (1.07– 2.78)	19.4 (2.66)	1.15 (0.74–1.79)

**Abbreviations:** CI = confidence interval; dB = decibel; OR = odds ratio; SE = standard error.

* An audiometric notch is a deterioration in the hearing threshold (the softest sound a person can hear). An audiometric notch is present when one or more of the thresholds at 3–4, or 6 kHz exceeds the pure-tone average of the 0.5 and1 kHz thresholds by 15 dB hearing level (HL) or more, and the 8 kHz threshold is at least 5 dB HL lower (better) than the highest threshold in the 3–6 kHz range. Audiograms were not accepted if the test and retest results were greater of 10 dB. The average 1-kHz frequency was the value used in this study. Participants were excluded if they had partial audio exam, ear compliance ≤0.2mL or pressure more negative than -150 dekapascals (daPa) (normal air pressure is approximately equal on both sides of the tympanic membrane [zero daPa]).

**^†^** Persons with no work exposure to noise included both those who reported off-work exposure to noise (e.g. noise from power tools, lawn mowers, farm machinery, cars, trucks, motorcycles, motor boats or music for 10 or more hours a week) and those who did not report exposure to off-work noise. Persons with work exposure to noise reported exposure to loud or very loud noise at work.

^§^ Participants were asked: “Which statement best describes your hearing (without a hearing aid)? Would you say your hearing is excellent, good, that you have a little trouble, moderate trouble, a lot of trouble, or are you deaf?”

Differences were identified by age, sex, and race/ethnicity, and by whether participants were exposed to loud noise at work. The presence of an audiometric notch increased with age (p<0.01), ranging from 19.2% among persons aged 20–29 years to 27.3% among persons aged 50–59 years (Table 1). The prevalence of notches was consistently higher in males than in females for both reported work exposure to noise and for no reported work exposure to noise (Table 2). This was true for both unilateral and bilateral notches (Figure).

**TABLE 2:** Percentages of adults aged 20–69 years with an audiometric notch* in one ear (unilateral notch) or both ears (bilateral notch), by reported work exposure to noise status,^†^ and selected characteristics — National Health and Nutrition Examination Survey, United States, 2011–2012

Characteristic	No reported work exposure to noise (n = 2,360)				Work exposure to noise (n = 1,223)			
	Bilateral or unilateral notch		Bilateral notch	Unilateral notch	Bilateral or unilateral notch		Bilateral notch	Unilateral notch
	% (SE)	OR (95% CI)	% (SE)	% (SE)	% (SE)	OR (95% CI)	% (SE)	% (SE)
**Overall**	**19.9 (2.04)**	**—**	**5.1 (0.73)**	**14.8 (1.53)**	**32.6 (2.48)**	**—**	**8.2 (1.09)**	**24.44 (2.20)**
**Sex**								
Male	24.7 (2.61)	Referent	7.2 (1.22)	17.6 (2.20)	39.1 (2.24)	Referent	10.2 (1.24)	28.9 (2.18)
Female	16.6 (2.17)	0.61 (0.44–0.83)	3.7 (0.89)^§^	12.9 (1.53)^§^	18.3 (3.65)	0.35 (0.22–0.55)	3.7 (1.99)^§^	14.6 (3.52)^§^
**Age group (yrs)**								
20–29	17.6 (2.91)	Referent	3.6 (1.69)	14.0 (1.78)	22.9 (5.07)	Referent	5.7 (2.21)	17.2 (4.67)^§^
30–39	18.6 (2.82)	1.07 (0.65–1.77)	3.4 (0.94)	15.2 (2.47)	37.3 (4.97)	2.00 (1.15–3.47)	6.9 (1.34)	30.4 (4.91)^§^
40–49	25.0 (3.74)	1.56 (0.91–2.68)	7.9 (1.95)	17.1 (3.05)	36.0 (3.46)	1.90 (1.05–3.43)	7.4 (2.37)	28.7 (1.66)
50–59	20.3 (3.04)	1.19 (0.73–1.95)	6.1 (1.43)	14.2 (2.46)	35.8 (2.73)	1.88 (1.01–3.50)	11.8 (2.60)	24.0 (2.87)
60–69	17.7 (3.06)	1.01 (0.59–1.72)	4.5 (1.12)	13.2 (2.91)	27.3 (5.23)	1.26 (0.60–2.66)	7.34 (1.57)	19.92 (5.37)
**Race/Ethnicity**								
White, non-Hispanic	19.4 (2.85)	Referent	5.1 (0.95)	14.3 (2.19)	31.9 (2.60)	Referent	8.7 (1.50)	23.2 (2.24)
Black, non-Hispanic	17.7 (1.47)	0.89 (0.58–1.38)	3.3 (0.56)	14.4 (1.40)	28.6 (2.62)	0.86 (0.64–1.14)	4.2 (1.14)^§^	24.4 (2.79)
Mexican American	24.2 (3.86)	1.39 (0.85–2.29)	8.7 (2.81)	14.8 (1.53)	43.0 (4.45)	1.61 (1.16–2.28)	14.8 (3.16)^§^	28.2 (3.58)^§^
**Education**								
Less than high school	22.0 (2.78)	1.17 (0.81–1.69)	8.2 (1.80)^§^	13.8 (2.41)	37.6 (6.52)	1.50 (0.89–2.53)	8.0 (3.26)	29.5 (5.43)
Completed high school	20.7 (3.70)	1.08 (0.73–1.61)	5.7 (1.65)	15.0 (2.87)	37.6 (4.06)	1.50 (0.98–2.32)	11.6 (2.42)§	26.0 (3.01)
More than high school	19.4 (2.07)	Referent	4.5 (0.85	14.9 (1.62)	28.6 (2.45)	Referent	6.7 (1.01)	21.9 (2.24)
**Poverty income ratio**								
≤1	21.2 (1.76)	1.27 (0.94–1.72)	4.2 (0.78)	17.0 (1.56)	25.9 (3.24)	0.81 (0.33–1.99)	7.3 (1.60)	18.5 (3.22)
>1 to <5	20.8 (3.04)	1.24 (0.93–1.65)	5.2 (1.18)	15.6 (2.29)	35.8 (2.65)	1.30 (0.61– 2.77)	8.8 (2.05)	27.0 (2.23)
≥5	17.5 (1.64)	Referent	5.7 (1.27)	11.8 (1.80)	30.1 (7.64)	Referent	7.7 (3.41)	22.4 (7.87)
**Self-reported hearing status^¶^**								
Excellent or good	20.1 (2.25)	Referent	5.0 (0.77)	15.0 (1.68)	31.0 (3.24)	Referent	6.6 (1.22)	24.4 (2.99)
Little, moderate, or lot of trouble	18.9 (3.94)	0.93 (0.51–1.69)	5.4 (2.26)	13.4 (3.10)	36.8 (2.36)	1.30 (0.93–1.81)	12.1 (2.01)^§^	24.7 (2.69)

**Abbreviations:** CI = confidence interval; dB = decibel; OR = odds ratio; SE = standard error.

* An audiometric notch is a deterioration in the hearing threshold (the softest sound a person can hear). An audiometric notch is present when one or more of the thresholds at 3, 4, or 6 kHz exceeds the pure-tone average of the 0.5 and1 kHz thresholds by ≥15 dB hearing level (HL), and the 8 kHz threshold is at least 5 dB HL lower (better) than the highest threshold in the 3–6 kHz range. Audiograms were not accepted if the test and retest results were greater of 10 dB. The average 1-kHz frequency was the value used in this study. Participants were excluded if they had partial audio exam, ear compliance ≤0.2mL or pressure more negative than -150 dekapascals (daPa) (normal air pressure is approximately equal on both sides of the tympanic membrane [zero daPa]).

**^†^** Persons with no work exposure to noise included both those who reported off-work exposure to noise (e.g. noise from power tools, lawn mowers, farm machinery, cars, trucks, motorcycles, motor boats or music for 10 or more hours a week) and those who did not report exposure to off-work noise. Persons with work exposure to noise reported exposure to loud or very loud noise at work.

^§^ Statistical difference at p<0.5 compared with the referent group.

^¶^ Participants were asked: “Which statement best describes your hearing (without a hearing aid)? Would you say your hearing is excellent, good, that you have a little trouble, moderate trouble, a lot of trouble, or are you deaf?”

**FIGURE:**
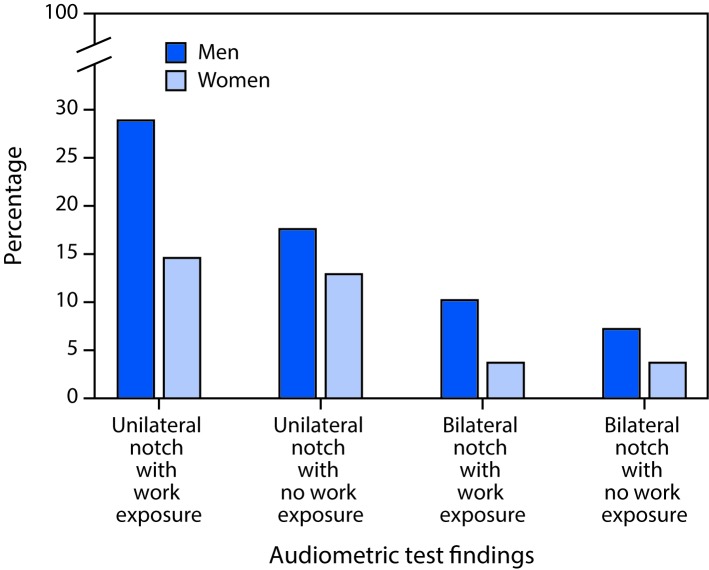
Percentage of persons with unilateral (in one ear) and bilateral (both ears) audiometric notches* in audiograms among adults aged 20–69 years who reported exposure to loud or very loud noise at work^†^ and those who reported no noise exposure at work, by sex — National Health and Nutrition Examination Survey, United States, 2011–2012 * An audiometric notch is a deterioration in the hearing threshold (the softest sound a person can hear). ^†^ Persons with no noise exposure at work included both persons who reported off-work exposure to noise (e.g., noise from power tools, lawn mowers, farm machinery, automobiles, trucks, motorcycles, motor boats, or music for 10 or more hours a week) and persons who did not report exposure to off-work noise.

Twenty-one million U.S. adults (19.9%) who reported no exposure to loud or very loud noise at work had an audiometric notch (bilateral or unilateral) (Table 1). Persons exposed to loud noise at work were twice as likely to have bilateral or unilateral notches (Table 1) than those not exposed. However, 23.5% of persons who self-reported excellent or good hearing (irrespective of noise exposure reported) had bilateral or unilateral notches (5.5% and 18.0%, respectively) (Table 1). These numbers were higher (31.0%) among persons reporting exposure to noise at work and lower (20.1%) among those who were not exposed to noise at work (Table 2). Seventy percent of persons exposed to loud noise in the past 12 months never or seldom wore hearing protection.

## Conclusions and Comments

Noise-induced hearing loss is a significant health problem among U.S. adults, is more prevalent among males, and increases with age. Persons with auditory damage caused by noise frequently do not recognize it; one in four U.S. adults who reported excellent or good hearing had an audiometric notch. Among persons who reported work exposure to loud noise, one third had a bilateral or unilateral notch.

Noise exposure is the second most common cause of acquired hearing loss (after aging) (*8*). An estimated 24% of hearing loss in the United States has been attributed to workplace exposures (*9*). Noise exposure is associated with numerous adverse health effects, and reducing noise exposure is likely to improve health. A recent study suggested that reducing environmental noise exposure might save lives by decreasing the prevalence of cardiovascular heart disease (*10*). Avoiding exposure to loud environments and effective use of personal hearing protection devices (earplugs or earmuffs) have been shown to prevent hearing loss (*3*). Evidence also exists that stronger occupational regulation leads to decreased noise levels (*11*). Persons who already have impaired hearing from noise exposure can benefit from clinical rehabilitation, such as amplification through hearing aids, learning to read lips, and other compensation strategies (*2*). Use of technology, such as smart phone apps to measure sound level, provides new ways of informing decisions and actions.^§^

Noise reduction and avoidance can prevent hearing loss or slow its progression. This can be accomplished by avoiding high volumes on personal listening devices; reducing listening time to high volumes of music; taking breaks from exposure; requesting lower volumes in public settings (restaurants, movie theaters); using quieter products (e.g., household appliances, power tools, recreational vehicles); reducing equipment noise by replacing worn or unbalanced machine parts; moving as far as possible from the loudest sound-producing source, such as loudspeakers or cannons at college stadiums; and using hearing protection devices (*2*,*3*). Hearing protectors need to fit well to reduce noise exposure effectively.

Noise exposure at younger ages needs particular attention. Damage to hearing accumulates over time so that hazardous exposure that begins earlier in life has the potential to be more damaging as persons age. The high prevalence of audiometric notches (one in five) among persons aged 20–29 years suggests that early life interventions need to be developed.

Hearing screenings can help reduce delays in diagnosis and improve access to hearing aids for those with hearing loss, thus improving health-related quality of life (*12*), yet a 2014 report found that only 46.0% of adults who had any trouble hearing had seen a health care professional about their hearing in the past 5 years (*5*). Hearing loss often progresses for years before being self-perceived or diagnosed (*13*,*14*). Talking to one’s personal health care provider about hearing loss symptoms, tests, and ways to protect hearing, might support early diagnosis and access to hearing rehabilitation if needed.

During routine exams, primary care providers can examine patients’ hearing; ask about patients’ hearing and noise exposures and inform them about the benefits of hearing protection; monitor patients with hearing loss symptoms, recommend or provide hearing tests when indicated; and counsel patients with hearing loss (*2*,*8*,*15*). Studies indicate, however, that 40%–77% of primary care providers have not asked about or screened for hearing loss (*16*,*17*). Patients reporting hearing-related symptoms (*15*) or risk factors such as noise exposure need to be referred for objective hearing assessment.^¶^^,^** Although there is currently a lack of data to support the benefits of regular hearing screening in adults aged >50 years, the American Speech-Language-Hearing Association^††^ recommends that adults be screened at least every decade through age 50 years and every 3 years thereafter. *Healthy People 2020*^§§^ includes objectives to increase the proportion of adults who have had a hearing examination in the past 5 years and to increase the number referred by their health care provider for hearing evaluation and treatment.

Although there are no federal regulations regarding exposure to nonoccupational noise, a 1974 Environmental Protections Agency report^¶¶^ identified 70 dB over 24 hours (75 dB over 8 hours) as the average exposure limit for intermittent environmental noise. World Health Organization (WHO) 1999 Guidelines for Community Noise*** recommend avoiding noise exposure levels that exceed 70 dB(A)^†††^ over a 24-hour period or 85 dB(A) over a 1-hour period. CDC’s National Institute for Occupational Safety and Health (NIOSH) has established an 8-hour, time-weighted average 85 dB(A) recommended exposure limit to protect most workers from developing hearing loss from noise exposure over a 40-year career. However, at that sound pressure level [85 dB(A) time-weighted average], approximately 8% of workers could still develop hearing loss, and thus NIOSH recommends that hearing protection be worn whenever noise levels exceed 85 dB(A), regardless of the length of exposure.

The U.S. Department of Health and Human Services (DHHS) and WHO are raising awareness about noise-induced hearing loss. DHHS is collecting data on hearing status and risk factors, as well as developing guidelines on hearing aids. WHO is developing guidelines on noise exposure. Other public entities, such as states and counties, partner with community groups to reduce noisy environments and use evidence to inform policies that decrease noise exposures. Other ways to reduce environmental noise exposure include using sound-absorbent materials in office buildings and public venues, erecting highway barriers, and passing noise ordinances. Managers and owners of public venues can decrease the loudest sound levels at those locations to help decrease noise exposure.

## Study Limitations

The findings in this report are subject to at least two limitations. First, this is a report of audiometric notches as a proxy for noise-induced hearing loss, and it is possible that some of the hearing loss observed through this method could be caused by factors other than noise. Second, establishing prevalence rates of hearing damage attributed to risk factors such as noise is confounded by multiple data limitations, such as reliance on self-reported rather than measured noise exposures, complexity of categorizing hearing loss; and co-occurrence of risk factors, including genetic predisposition, and aging.

## Data Needs

This study examines evidence of hearing loss related to noise exposure in a single NHANES 2-year data cycle. It does not provide a longitudinal assessment of persons over time, nor does it compare the results of hearing examinations across different NHANES cycles. A need exists for longitudinal data that measure cumulative effects of noise exposure on hearing over time. These data also show high prevalence of audiometric notches in young adults. Recent studies have shown an increase in the number of young persons exposed to loud noise and music via personal listening devices and at entertainment venues. Future work is needed on early life exposure to noise and its relation to hearing later in life.

**Key Points**

• Noise exposure at home and in the community can permanently damage hearing.

• Almost one in four adults who reported excellent to good hearing already have measurable hearing loss.

• The presence of noise-induced hearing loss increased from one in five among young adults aged 20–29 years to one in four among adults aged 50–59 years.

• Additional information is available at https://www.cdc.gov/vitalsigns/.

## Footnotes

* National Health Interview Survey, 2014 data. https://www.cdc.gov/mmwr/preview/mmwrhtml/mm6437a8.htm.

† https://www.cdc.gov/nchs/nhanes/.

§ https://blogs.cdc.gov/niosh-science-blog/2014/04/09/sound-apps/.

¶ U.S. Preventive Services Task Force. Report no. 11-05153-EF-1. Hearing loss in older adults: screening. https://www.uspreventiveservicestaskforce.org/Page/Document/RecommendationStatementFinal/hearing-loss-in-older-adults-screening.

** NIOSH. Criteria for a recommended standard — occupational noise exposure: revised criteria. Publication No. 98–126. https://www.cdc.gov/niosh/docs/98-126/pdfs/98-126a.pdf.

†† http://www.asha.org/uploadedFiles/aud/InfoSeriesAudScreen.pdf.

§§ https://www.healthypeople.gov/2020/topics-objectives/topic/physical-activity/objectives?topicId=33.

¶¶ https://nepis.epa.gov/Exe/ZyPDF.cgi/2000L3LN.PDF?Dockey=2000L3LN.PDF.

*** http://www.euro.who.int/en/health-topics/environment-and-health/noise/publications.

††† dBA indicates A-weighted decibels, an expression of the relative loudness of sounds in air as perceived by the human ear. In the A-weighted system, the decibel values of sounds at low frequencies are reduced, compared with unweighted decibels, in which no correction is made for audio frequency.

## References

[1] Blackwell DL, Lucas JW, Clarke TC. Summary health statistics for U.S. adults: national health interview survey, 2012. Vital Health Stat 10 2014;260:1–161. 24819891

[2] National Academies of Sciences, Engineering, and Medicine. Hearing health care for adults: priorities for improving access and affordability. Washington, DC: The National Academies Press; 2016. 27280276

[3] Themann CL, Suter AH, Stephenson MR. National research agenda for the prevention of occupational hearing loss—part 1. Semin Hear 2013;34:145–207. 10.1055/s-0033-1349351

[4] Stucky SR, Wolf KE, Kuo T. The economic effect of age-related hearing loss: national, state, and local estimates, 2002 and 2030. J Am Geriatr Soc 2010;58:618–9. 10.1111/j.1532-5415.2010.02746.x 20398138

[5] Zelaya CE, Lucas JW, Hoffman HJ. Self-reported hearing trouble in adults aged 18 and over: United States, 2014. NCHS Data Brief 2015;214:1–8 https://www.cdc.gov/nchs/products/databriefs/db214.htm.https://www.ncbi.nlm.nih.gov/entrez/query.fcgi?cmd=Retrieve&db=PubMed&list_uids=26462204&dopt=Abstract 26462204

[6] Basner M, Babisch W, Davis A, Auditory and non-auditory effects of noise on health. Lancet 2014;383:1325–32. 10.1016/S0140-6736(13)61613-X 24183105 PMC3988259

[7] Hoffman HJ, Ko CW, Themann CL, Dillon CF, Franks JR. Reducing noise-induced hearing loss (NIHL) to achieve US healthy people 2010 goals [Abstract]. Am J Epidemiol 2006;163:S122.

[8] Rabinowitz PM. Noise-induced hearing loss. Am Fam Physician 2000;61:2749–56, 2759–60. 10821155

[9] Tak S, Calvert GM. Hearing difficulty attributable to employment by industry and occupation: an analysis of the National Health Interview Survey—United States, 1997 to 2003. J Occup Environ Med 2008;50:46–56. 10.1097/JOM.0b013e3181579316 18188081

[10] Gan WQ, Davies HW, Koehoorn M, Brauer M. Association of long-term exposure to community noise and traffic-related air pollution with coronary heart disease mortality. Am J Epidemiol 2012;175:898–906. 10.1093/aje/kwr424 22491084

[11] Verbeek JH, Kateman E, Morata TC, Dreschler WA, Mischke C. Interventions to prevent occupational noise-induced hearing loss: a Cochrane systematic review. Int J Audiol 2014;53(Suppl 2):S84–96. 10.3109/14992027.2013.857436 24564697 PMC4678960

[12] Chisolm TH, Johnson CE, Danhauer JL, A systematic review of health-related quality of life and hearing aids: final report of the American Academy of Audiology Task Force On the Health-Related Quality of Life Benefits of Amplification in Adults. J Am Acad Audiol 2007;18:151–83. 10.3766/jaaa.18.2.7 17402301

[13] Le Prell CG, Hensley BN, Campbell KC, Hall JW 3rd, Guire K. Evidence of hearing loss in a ‘normally-hearing’ college-student population. Int J Audiol 2011;50(Suppl 1):S21–31. 10.3109/14992027.2010.540722 21288064 PMC3095511

[14] Rota-Donahue C, Levey S. Noise-induced hearing loss in the campus. Hear J 2016;69:38–9. 10.1097/01.HJ.0000484551.28667.81

[15] Walling AD, Dickson GM. Hearing loss in older adults. Am Fam Physician 2012;85:1150–6. 22962895

[16] Wallhagen MI, Pettengill E. Hearing impairment: significant but underassessed in primary care settings. J Gerontol Nurs 2008;34:36–42. 10.3928/00989134-20080201-12 18286791

[17] Cohen SM, Labadie RF, Haynes DS. Primary care approach to hearing loss: the hidden disability. Ear Nose Throat J 2005;84:26–44. 15742769

